# Malaria risk and access to prevention and treatment in the paddies of the Kilombero Valley, Tanzania

**DOI:** 10.1186/1475-2875-7-7

**Published:** 2008-01-09

**Authors:** Manuel W Hetzel, Sandra Alba, Mariette Fankhauser, Iddy Mayumana, Christian Lengeler, Brigit Obrist, Rose Nathan, Ahmed M Makemba, Christopher Mshana, Alexander Schulze, Hassan Mshinda

**Affiliations:** 1 Department of Public Health and Epidemiology, Swiss Tropical Institute, PO Box, CH-4002 Basel, Switzerland; 2 Ifakara Health Research and Development Centre, PO Box 53, Ifakara, Tanzania; 3 Novartis Foundation for Sustainable Development, WRO-1002.11.56, CH-4002 Basel, Switzerland

## Abstract

**Background:**

The Kilombero Valley is a highly malaria-endemic agricultural area in south-eastern Tanzania. Seasonal flooding of the valley is favourable to malaria transmission. During the farming season, many households move to distant field sites (*shamba *in Swahili) in the fertile river floodplain for the cultivation of rice. In the *shamba*, people live for several months in temporary shelters, far from the nearest health services. This study assessed the impact of seasonal movements to remote fields on malaria risk and treatment-seeking behaviour.

**Methods:**

A longitudinal study followed approximately 100 randomly selected farming households over six months. Every household was visited monthly and whereabouts of household members, activities in the fields, fever cases and treatment seeking for recent fever episodes were recorded.

**Results:**

Fever incidence rates were lower in the *shamba *compared to the villages and moving to the *shamba *did not increase the risk of having a fever episode. Children aged 1–4 years, who usually spend a considerable amount of time in the *shamba *with their caretakers, were more likely to have a fever than adults (odds ratio = 4.47, 95% confidence interval 2.35–8.51). Protection with mosquito nets in the fields was extremely good (98% usage) but home-stocking of antimalarials was uncommon. Despite the long distances to health services, 55.8% (37.9–72.8) of the fever episodes were treated at a health facility, while home-management was less common (37%, 17.4–50.5).

**Conclusion:**

Living in the *shamba *does not appear to result in a higher fever-risk. Mosquito nets usage and treatment of fever in health facilities reflect awareness of malaria. Inability to obtain drugs in the fields may contribute to less irrational use of drugs but may pose an additional burden on poor farming households. A comprehensive approach is needed to improve access to treatment while at the same time assuring rational use of medicines and protecting fragile livelihoods.

## Background

Malaria continues to be a major public health problem, particularly for young children in sub-Saharan Africa [1]. Malaria has often been linked with agricultural practices, especially irrigation. Environmental management, such as well-designed irrigation schemes have been proposed as one of the means for reducing the burden of malaria in agricultural settings [2,3].

Research on malaria risk related to farming activities has so far mainly focused on vector abundance and malaria transmission in areas with artificial irrigation [4-6]. Linkages between malaria and agriculture were found to be complex and specific to the local setting. Cultivation of rice may encourage the proliferation of malaria-transmitting *Anopheles *mosquitoes [6,7]. Yet, due to lower density of human hosts in the fields in some areas, or zoophilic feeding behaviour of some vector species in others [8], an increase in the number of mosquitoes does not necessarily translate into higher parasite prevalence in the human population. Research done in northern Tanzania reported lower malaria transmission in villages with irrigated crop production compared to adjacent savannah villages without irrigation. This was attributed to a higher standard of living and better health care in the studied irrigation schemes and has been referred to as the "paddies paradox" [4].

By contrast, an analysis of the spatiotemporal patterns of malaria transmission in Thailand provided some evidence that the farming season and the movement of people to the location of their crops were associated with higher risk of malaria [9]. Socio-cultural aspects were investigated in a study in Côte d'Ivoire which indicated that socio-economic transformations and changes in gender-specific tasks and responsibilities induced by the intensification of inland valley rice cultivation lead to a reduction of the capacity of women to manage disease episodes [10]. Overall, no simple and clear association has so far been found between irrigated rice cultivation and malaria transmission [11].

The Kilombero Valley in south-eastern Tanzania is a well-described highly malaria-endemic tropical wetland dominated by subsistence agriculture [12-15]. The valley's climatic and ecological characteristics are favourable for high and perennial malaria transmission. During the rainy season, large parts of the valley are flooded by the Kilombero River. The majority of the valley's farming residents take advantage of this natural flood irrigation to cultivate rice, which is the main staple food and the most important cash crop in the area. Only very few artificial irrigation schemes are in place. While artificial schemes may be established based on knowledge of water management and mosquito ecology [3], there is little opportunity to influence the natural flooding of a vast floodplain such as the Kilombero Valley.

**Figure 1:**
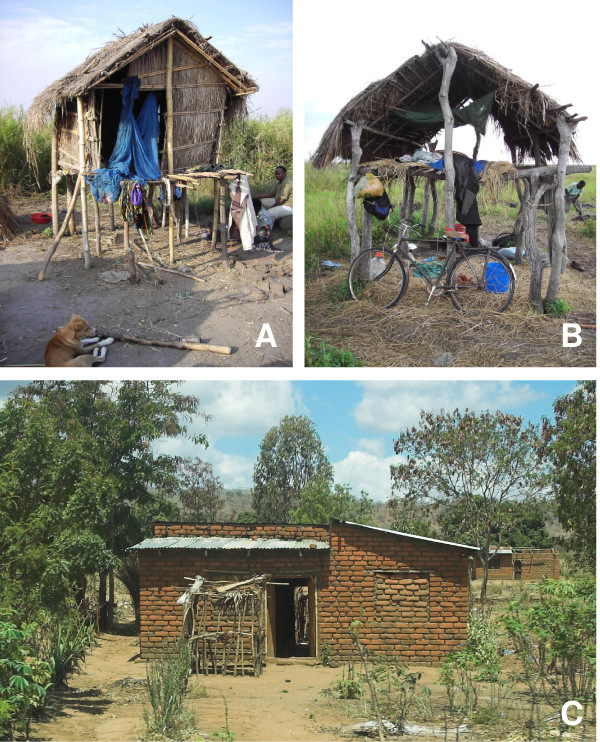
*Shamba *houses (A & B) and main house in a village (C).

The geographical and ecological patterns of the Kilombero Valley lead to distinct seasonal movements of the local farming population. The valley's villages with their entire infrastructure are situated along the main roads on the edges of the flood plain. All health care providers, such as health facilities, drug stores or general shops selling drugs are located in the villages. Farming sites (*shamba *in Swahili) are often located at a considerable distance from people's homes in the fertile lower wetland. Due to the standing water, farms can often only be accessed by walking long distances and wading through water. During the main cultivation period, families often move from the villages to the farms where they stay in temporary shelters, usually fabricated with branches and straw and often built on stilts to protect them from water and wild animals (Figure 1a and 1b).

The rainy season and hence main cultivation period occurs between February and June. It is considered a period of high vulnerability for the farming population because of recurring food insecurity (empty stocks before the harvest), labour stress (intensive work on the farm), poor access to preventive and curative health services as well as family support due to the remoteness of the farms, little time for child care and poor access to clean water and adequate sanitation in the fields. Malaria transmission, fever incidence and mortality in the community show seasonal variations with a peak during the same season. It has, therefore, been speculated that the seasonal movement of parts of the population to their distant fields results in an increased risk of and vulnerability to malaria. Long distances from the farming sites to the next village and health facility can furthermore contribute to delayed and inappropriate care-seeking which may in part be compensated by more frequent use of home-stocked drugs. Eventually, disease episodes during the cultivation period may result in lower crop yield and consequently lower income with considerable impact on household economies, as was shown in a study conducted in Côte d'Ivoire [16].

Hardly any of the literature published so far has focused on the impact of these seasonal movements on malaria incidence or the ability of people to cope with disease episodes, and it is unclear, how common such movement patters are on the African continent. The research presented here provides a qualitative and quantitative assessment of risks, access to health services and treatment-seeking related to fever episodes that occurred in farming households during the main cultivation period.

## Methods

This longitudinal study was carried out between January and August 2005 within the frame of a research and intervention project to understand and improve access to effective malaria treatment (ACCESS Programme), which is described in detail elsewhere [17].

### Study area and study population

The study was conducted in the area of a Demographic Surveillance System (DSS), in the districts of Kilombero and Ulanga, Tanzania. In mid-2005, the DSS had a population of 75'120. There is a short rainy season from October to December and a long one from February to June. Annual rainfall ranges from 1,200 to 1,800 mm and annual mean temperature is around 26°C [18]. The details of the study area have been described elsewhere [17].

**Figure 2:**
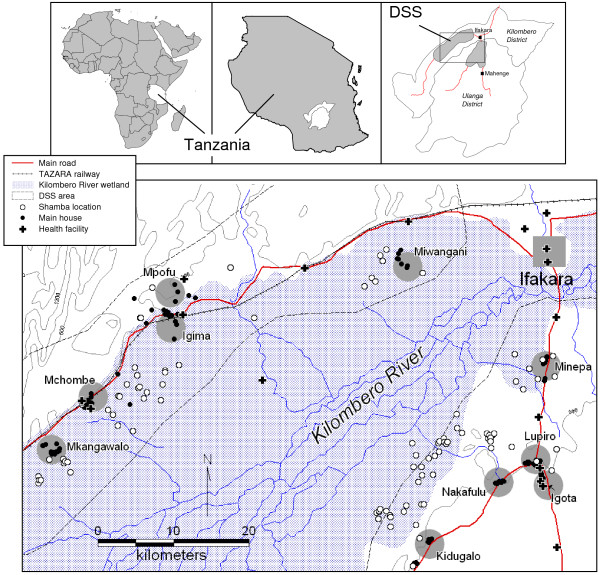
Study area with Demographic Surveillance System (DSS), ten sampled villages (villages centres shaded grey), main houses and *shamba *locations.

For the initial visit in January 2005, ten out of a total of 25 DSS villages were chosen at random (Figure 2). Out of 5,912 households that had previously reported to have a farming plot (*shamba*), a two-stage random sample of 159 households was drawn, proportional to the relative size of the villages. To compensate for 77 (54.7%) households which reported not to have a *shamba *anymore in 2005, an additional sample of 80 households was added to the study in March using the same sampling strategy. Of these, another 40 (50%) households did not have a *shamba *anymore or did not cultivate it that year. The total number of households visited per round is reported in Table 1.

**Table 1:** Number of households (main residencies and *shamba *houses) visited in each round

**Round**	**Time**	**Main houses**	**Shamba**	Total**
1	15–29 Jan	72	N/A	72
2	2–20 March	25	47	72
2b*	29 Mar–4 Apr	40	N/A	40
3	6–27 Apr	35	67	102
4	12–23 May	32	69	104
5	11–27 Jun	28	69	98
6	23 Jul–1 Aug	61	26	87

* additional sample to compensate for losses to follow-up, as explained in the text

** in round 4 and 5, the differences between the sum of the two locations and the total value are due to missing information on the interview location

N/A = Not applicable

### Data collection

Data was collected monthly from all households included in the study. Baseline data collection started in January 2005 in the main residences in the villages. The subsequent visits (rounds) took place in the *shamba *unless all family members were in the main residence at the time of the monthly visit. The sixth and last visit was done in July 2005 or earlier if the family had completely moved back from the fields to their main house. At every visit, two data collection tools were used:

#### Household-level questionnaire

During each round, the same structured questionnaire was applied to record each household member's whereabouts in the four preceding calendar weeks (day-time and night-time), activities performed on the farms, use of mosquito nets and number of recent fever episodes. The location of the main house and the *shamba *were recorded using hand-held GPS devices (Garmin^® ^e-Trex^®^, Garmin Ltd.).

#### Treatment-seeking questionnaire (EMIC)

For those fever episodes which had occurred in the preceding two weeks, an in-depth interview was carried out to elicit detailed information on treatment seeking. Interviews were done with patients of 12 years and above and with caretakers of children under five years of age. Cases that had not recovered the day of the interview were not interviewed and advised to seek care at a health facility. An Explanatory Model Interview Catalogue (EMIC) was used for data collection. EMICs are semi-structured questionnaires designed to collect quantitative and qualitative information (narratives) on the experience of illness and resulting treatment-seeking behaviour from the point of view of those affected [19]. The EMIC was developed based on preceding focus-group discussions [17] and further qualitative research on people's understanding of malaria [20-22]. The same forms had already been used in a previous treatment-seeking survey in the same area [23].

In addition, information on the socioeconomic ranking of the households in the study was obtained from the DSS database. Each year, information is collected on proxy markers of socioeconomic status such as main sources of income, household ownership of assets, housing characteristics and water and sanitation facilities for all of the households in the DSS area. The 2005 survey collected information on 15,396 households.

### Data entry and analysis

Data was double-entered at the Ifakara Health Research and Development Centre (IHRDC) data unit using Microsoft Access or FoxPro software (Microsoft Corp., Seattle, USA). Statistical analysis was done in Intercooled Stata 9 (StataCorp, College Station, Texas, USA) and mapping using MapInfo Professional 7.0 (MapInfo Corp., Troy, New York, USA) and ArcView GIS 3.3 (ESRI, Redlands, CA, USA).

An individual level and a household level model were fitted to quantify the effect of risk factors for a fever episode.

A logistic model was fitted at the individual level to quantify the effect of age and proportion of weeks spent in the *shamba *overnight on fever incidence. The outcome variable at individual level was derived from the EMIC interviews. As EMICs were only done for recovered episodes (excluding acute cases), the outcome indicator represented only a proportion of the overall fever incidence. In the five cases in which more than one episode was recorded on the same person, one episode was chosen at random. At the household level, a logistic model with outcome variable "reported fever episode in the household in the past two weeks" was fitted to quantify the effect of household mosquito net ownership and use. In the final individual and household models, no account was taken of the clustering within individuals and households. Comparing the models with and without clustering using the likelihood ratio test revealed no evidence of significant between-individual or between-household variance.

A descriptive analysis of household data on net ownership and drug home stocking was carried out. Averages were calculated over all rounds with no weighting since in each round approximately the same number of households was interviewed. Net usage average was weighed by number of observations (persons) per round.

Treatment-seeking indicators were assessed at an individual level using EMIC data. All estimates derived from this retrospective study relied on self-reported help-seeking behaviour. Logistic models were fitted to assess the effect of likely predictors on several treatment seeking indicators.

Principal components analysis (PCA) was used to define the weights of a relative index of socioeconomic status (SES). The first principal component was chosen, since it summarizes the largest amount of information common to the asset [24,25]. The index was constructed for 14,603 out of 15,396 (94.8%) households in the DSS area from the following dichotomous variables: living in a rented accommodation (10% of the households), having income from any sort of business (11%), ownership of bike (49%), radio (57%), chicken (59%), and other animals (4%); presence of iron roof (30%), concrete/brick wall (44%), cemented floor (12%), toilet (91%) and toilet wall (12%) in the house. In addition, the following variables were measured on an ordinal scale: hectares of land owned (5% had no land, 30% up to one hectare, 31% more than one and up to two hectares, 17% had more than two and up to three hectares and 17% had more than three hectares), number of rooms in the house (30% had one bedroom, 42% had two, and 28% had three or more) and number of mosquito nets owned (6% had none, 32% had one, 34% had two and 28% had three or more).

The first principal component accounted for 22% of the variability. Greatest weight was given to ownership of tin roof (0.39), the presence of concrete/brick walls (0.35), cemented floor (0.33), ownership of bikes (0.30), lighting by candles or petrol lamp (0.30), ownership of mosquito nets (0.29), number of bedrooms (0.28), wall around the toilet (0.27), and ownership of a radio (0.26). Households were classified into wealth-quintiles with mean indices of -2.25, -1.23, -0.32, 0.83, and 2.97. Households from the study were assigned their SES index and category from the analysis performed on the entire DSS database.

### Ethical consideration

Study participants were informed about the study framework, aim and purpose and oral informed consent was obtained. Village authorities were informed about the activities prior to their onset. The study was granted ethical clearance as part of the ACCESS Programme by the institutional review board of the IHRDC and the Tanzanian National Medical Research Coordinating Committee (NIMR/HQ/R.8a/Vol.IX/236).

## Results

### Sample characteristics

In each round, between 72 and 104 households were visited (Table 1). Over the six rounds 575 household visits were carried out and information was collected on 703 individuals. On average there were 5.92 (SD 2.46) members in each household. The sample comprised 24 infants (age <1 year), 82 children aged 1 to <5 years, 150 children aged 5 to <12 years, 66 adolescents aged 12 to <16 years and 354 adults (age 16 years and above). For 27 individuals no age was recorded. The age structure corresponded closely with the national age structure as reported in the 2002 population census [26]. The distribution of the study-households in SES quintiles was as follows: 16% poorest, 21% second poorest, 26% middle, 23% second richest, 14% richest.

### Exposure in the shamba

The field sites were on average located at a median linear distance of 7.3 km (interquartile range [IQR] 3.7–12.1) from the main houses. Main houses were closer to the nearest health facility (median distance 2.2 km; IQR 0.8–5.6) than *shamba *locations (7.9 km; IQR 6.0–10.1) (Wilcoxon rank-sum test W = -9.28, P < 0.001). Actual travel distances and times between main house and *shamba *or to the next health facility did not only depend on the distance, but also on the condition of the trails, the available means of transport (such as motorbike, bicycle, or canoe), and water levels. This was reflected in a parallel study in the same area in which distances to the next health facility of up to 19 km along people's walking paths were measured [27].

**Figure 3:**
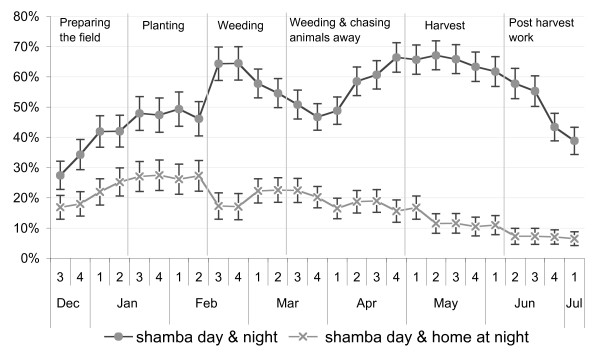
Percentage of members of visited households in the *shamba *each week.

**Figure 4:**
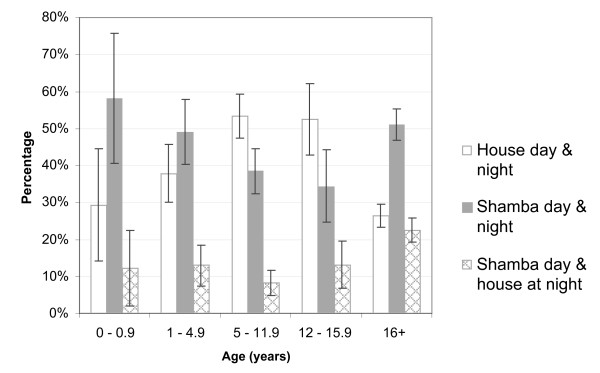
Percentage of time (weeks) spent in the *shamba *or at home over entire study period. Error bars are 95% confidence intervals.

Analysis of the reported whereabouts of people each week showed that depending on the activities performed in the fields, large parts of the households moved to the *shamba *for overnight stays. During the weeding (mid-February to mid-March) and harvesting seasons (May), the proportion of household members who spent days and nights in the fields peaked. Throughout the study period, going to the *shamba *only for the day was less frequent than staying overnight, which reflects the long distances and difficult accessibility of the field sites. However, at the beginning of the farming season when the crops were planted (January – mid-February) a higher percentage of household members only spent the day in the fields and moved back to their main residence overnight (Figure 3). This proportion decreased in the following months. Over the whole study period, adults (over 16 years) and children below five years were most frequently found to spend the nights in the *shamba*, while children between 5–16 years (i.e. about school age) were most frequently permanently in the main house (Figure 4). Over the whole study, people spent, on average, 40.0% of the weeks in the *shamba *overnight.

### Preventive measures

Overall, coverage and usage rates of mosquito nets were extremely high. On average, there was a mosquito-net in 93.6% of the main houses (95% confidence interval [CI] 89.9–96.2) and in 96.8% (93.9–98.5) of the *shamba *huts. Household ownership of nets treated with insecticide (ITN) was lower with 58.9% (52.7–64.9) and 59.2% (43.2–65.0) in the main house and the *shamba*, respectively. Averaged over the study period, 97.9% (95.2–100) of the people sleeping in the *shamba *reported to have used a mosquito net (treated or not) the preceding night.

**Figure 5:**
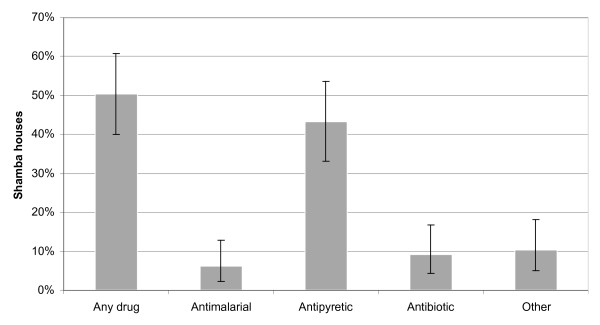
Drug home stocking in the *shamba *huts. Error bars are 95% confidence intervals.

Over the whole study period, 50.5% (40.2–60.8) of the households reported to stock drugs in their *shamba *hut, varying from 21.2% to 34.0% between the rounds. Antipyretics were found most frequently (43.3% of all households; 33.3–53.7), while only few households stocked antimalarial drugs (6.2%; 2.3–13.0) (Figure 5).

### Fever incidence

Over the study period 29% of households visited reported fever cases in the preceding two weeks. Some households (n = 27 over all rounds) reported more than one fever episode. At an individual level, averaged over the whole study period, this accounts for a two-week incidence rate of 6.1%, which is lower than the 13% (7,102/55,514) reported routinely from the DSS for that period (Table 2). In the rounds in which all visits took place at the main house (rounds 1 and 2b), the fever incidence was closer to the reported DSS average.

**Table 2:** Number of individuals and households visited in each round and reported two-week fever incidence

	**HH level**			**Individual level**		
	
**Round**	**N**	**Fever**		**N**	**Fever**	
				
		**%**	n**		**%**	n**
1	72	59	30 (/51)	390	13.9	41 (/293)
2	72	28	20	409	6.4	26
2b*	40	42	14 (/33)	209	8.7	17 (/195)
3	102	24	24 (/101)	584	4.7	32 (/596)
4	104	26	27	596	5.4	32
5	98	19	18	547	3.5	19 (/541)
6	87	29	26	489	5.5	27
Overall	575	29	158 (/545)	3224	6.1	189 (/3103)

* additional sample to compensate for losses to follow-up, as explained in the text

** numbers in brackets denote the denominator excluding missing values

In total, 58 of these fever cases were followed up with an EMIC interview. However, one person was followed up on three consecutive occasions and three individuals on two consecutive occasions, leaving 52 independent EMIC interviews for the analysis at individual level (27 persons of 12 years and above, and 25 children under five years of age).

### Risk factor analysis at individual level

Results from the univariate model (Table 3) fitted at individual level showed evidence of a very slight increase in the odds of fever for every 10% increase in weeks spent overnight in the shamba (OR = 1.09; 1.02–1.17). However, the effect was confounded by age and once this was accounted for in the multivariate model the effect of time spent in the *shamba *lost its significance. Age was a very strong independent predictor of fever incidence (likelihood ratio test [LRT] of inclusion chi-square = 34.62, P < 0.001). The odds of children between one and five years of age having a fever were 4.47 times the odds of adults (2.35–8.51). No evidence could be found of an increased risk for infants, although this may have been due to lower sample size in this age group. There was no evidence of an interaction between the effect of age and time spent at the *shamba *overnight (LRT of inclusion chi-square = 6.13, P = 0.101).

**Table 3:** Univariate and multivariate analyses of the relationship between fever incidence and risk factors (logistic regression).

**Risk factors**	**Univariate**			**Multivariate**		
	
	**n**	**Odds Ratio (95% CI)**	P*	**n**	**Adjusted OR (95%CI)**	P*
% weeks at *shamba *overnight (10% increase)	688	**1.09 (1.02 to 1.17)**	**0.013**	667	1.06 (0.98 to 1.14)	0.142

Age (years)	676					<0.001
0–0.9	24	2.63 (0.83 to 8.27)	0.098	23	2.51 (0.79 to 8.01)	0.119
1–4.9	82	**4.53 (2.38 to 8.58)**	**<0.001**	82	**4.47 (2.35 to 8.51)**	**<0.001**
5–11.9	150	0.64 (0.27 to 1.51)	0.317	150	0.71 (0.30 to 1.71)	0.448
12–15.9	66	0	0.026	65	0	n/a
16+	354	1	**-**	347	1	**-**

* Wald test of significance of effect, LLR test of significance of variable in the model

### Risk factor analysis at household level

Analysis of the crude effect of location of the interview showed a significant decrease in the odds of fever in households for which the interview was carried out at the *shamba *rather than in the main house (OR = 0.53, 0.36–0.77). However, once corrected for the number of households in each round this effect was longer significant. The analysis at the household level showed no association of reported fever cases and net ownership (treated or untreated) or use. Given the consistently high level of net use, this was not surprising. No association was found between number of household members and fever incidence. However, households with a higher SES score were found to be more likely to report a fever case (OR = 1.16, 1.01–1.32) (Table 4). This slight positive association may be attributed to a reporting bias. However, a chance finding cannot be ruled out as no association could be found when the same analysis was repeated for the entire DSS population (OR = 1.02, 0.76–1.36).

**Table 4:** Univariate analysis of the relationship between reported fever case in a household and household factors (logistic regression)

**Household risk factors**	**n**	Odds Ratio* (95% CI)	**P**
Interview at the shamba	542	0.79 (0.50 to 1.23)	0.300
Net ownership in the household	542	0.78 (0.34 to 1.79)	0.564
Number of nets owned	543	1.08 (0.89 to 1.30)	0.439
Number of treated nets	541	1.01 (0.86 to 1.19)	0.873
Number of people sleeping under a net	545	1.00 (0.94 to 1.06)	0.963
SES index	479	**1.16 (1.01 to 1.32)**	**0.032**
Number of household members	545	0.99 (0.91 to 1.06)	0.820

* adjusted for number of households in each round

### Treatment-seeking

In total, 90.4% (79.0–96.8) of the followed-up fever cases were treated with one or more of the recommended antimalarials (55.8% sulphadoxine-pyrimethamine [SP], 28.9% amodiaquine, 13.5% quinine). 25.0% (14.0–38.9) of these antimalarials were administered on the same day, 71.2% (56.9–82.9) on the same or the next day. 53.8% (39.5–67.8) attended a health facility during the course of their illness, while 36.5% (23.6–51.0) practised exclusive home-management with shop-bought or leftover drugs from home stocks.

There was no statistically significant difference in these indicators between children and adults or between socio-economic groups. Treatment with a recommended antimalarial within two days was found to be slightly more frequent in under-fives (84.0%, 63.9–95.5) than in adolescents and adults above five years of age (59.3%, 38.8–77.6), with borderline significance (P = 0.056). Whether the episode was first recognized in the *shamba *or the main house had no effect on whether it was treated with an antimalarial, treated within one or two days, or brought to a health facility (Table 5).

**Table 5:** Treatment indicators for fever episodes recognized at home and in the *shamba*

**Indicator**	**Home Percentage (95% CI)**	**Shamba Percentage (95% CI)**	**P**
N	22	30	

Treated with any drug	95.8% (78.9 to 99.9)	97.1% (84.7 to 99.9)	0.803
Antimalarial (AM)*	95.8% (78.9 to 99.9)	88.2% (72.6 to 96.7)	0.290
AM on day 1 or 2*	62.5% (40.6 to 81.2)	76.5% (58.8 to 89.3)	0.252
AM on day 1*	16.7% (4.7 to 37.4)	32.4% (17.4 to 50.5)	0.171
Health facility visit	58.3% (36.6 to 77.9)	55.8% (37.9 to 72.8)	0.853
Exclusive HMM^‡^	37.5% 18.8 to 59.4)	32.4% (17.4 to 50.5)	0.685

* Recommended antimalarial (AM): SP, amodiaquine or quinine

^‡ ^Home-management

In the multivariate analysis, longer distance to the nearest health facility seemed to be associated with less exclusive home management (OR = 0.79, 0.63–1.00) i.e. paradoxically, households whose *shamba *was far from a facility were less likely to administer an antimalarial without ever attending a health facility. No other significant predictors were found (Table 6). In reverse, visiting a health facility was slightly more likely if households were located further away from a facility (OR = 1.40, 1.03–1.89).

**Table 6:** Multivariate analysis of factors related to exclusive home management (logistic regression)

**Risk factors**	**Univariate**			**Multivariate**		
	
	**n**	**Odds Ratio (95% CI)**	**P**	**n**	**Adjusted OR (95% CI)**	**P**
Age group						
12+ years	27	1		19	1	
<5 years	25	0.68 (0.22–2.14)	0.514	24	0.49 (0.10–2.49)	0.391

Illness recognized						
Home	22	1		18	1	
*Shamba*	30	0.72 (0.23–2.26)	0.576	25	2.09 (0.36–12.04)	0.409

Type of closest provider						
Health facility	15	0.82 (0.23–2.90)	0.760	16	1	
General shop	18	0.36 (0.10–1.33)	0.126	15	0.33 (0.05–2.45)	0.281
Drug store	12	1.32 (0.35–4.96)	0.675	12	1.25 (0.18–8.79)	0.821

Distance to health facility (km)	42	**0.83 (0.68–1.00)**	**0.050**	**43**	**0.79 (0.63 to 1.00)**	**0.046**

Distance to nearest provider (km)	42	0.82 (0.69–0.98)	0.029	43	**-**	**-**

Location						
Kilombero	24	1		18	**-**	**-**
Ulanga	28	0.33 (0.10–1.08)	0.066	25		

SES score	47	0.93 (0.64–1.35)	0.694	43	0.79 (0.46–1.36)	0.395

* Wald test of significance of effect

The same analysis for non-exclusive home management (i.e. treatment with an antimalarial not obtained from a health facility, irrespective of whether a health facility was attended or not) as outcome did not reveal a significant effect of distance to the nearest health facility. However, non-exclusive home-management was more likely if the nearest provider was a drug store rather than a health facility or general shop (OR 8.95, 1.23–65.19). Hence, it appeared as if antimalarials would rather be obtained from outside a health facility if a drug store was close – but without preventing people from visiting a facility at some stage.

No significant predictors for administration of an effective antimalarial within one or two days were found in this analysis.

## Discussion

The seasonal movement of people to distant field sites is a prevailing pattern in the Kilombero Valley of Tanzania, and a major determinant of the lives of entire households. To which extent this pattern is common in the rest of sub-Saharan Africa is unclear, given the scarcity of data on this issue. Household members spend a considerable part of the farming season living in very basic shelters under difficult conditions. During the harvesting period, over 60% of the members of the farming households moved to the fields from where access to health services and other infrastructure is more difficult. Walking distances of up to 19 km are a reality in some areas [27]. Conversely, this also means that during the farming season a large part of the local population is difficult to reach with health services, messages and campaigns. Adults who bear the heaviest burden are those most frequently spending the nights in the *shamba *houses. Young children, who are most at risk of contracting malaria and generally more vulnerable, are often accompanying their parents and are, therefore, exposed to the same adverse conditions. Only school-aged children were spending most of the time in the villages, since school-attendance is compulsory for seven years.

Adverse conditions in the *shamba *are clearly recognized by the local population. Extremely high (98%) mosquito-net usage rates reflect the immense nuisance posed by mosquitoes as well as the success of health education and social marketing in promoting the use of ITNs. Social marketing for ITNs has been intensive in the study area since the implementation of an ITN programme in 1997 [28]. However, many mosquito nets are not treated with insecticide calling for interventions to increase re-treatment rates or the implementation of long-lasting insecticidal nets (LLIN). Preparedness for malaria illness episodes is rather poor with only 6% of the households stocking antimalarials in the *shamba*, which may to some extent reflect the lower availability of the first-line treatment SP in shops in the study area [29]. Better availability of appropriate antimalarial drugs close to people's homes could potentially promote prompt treatment of malaria episodes [30,31]. Currently, registered drug stores and health facilities do not reach the *shamba *locations and this is unlikely to rapidly. Alternative approaches to provide people in the *shamba *with appropriate treatment should therefore be explored.

While accessibility to treatment services is clearly more difficult from the *shamba*, spending time there did not increase the odds of having a fever overall. Fever rates observed in the *shamba *were even lower than those in the main houses or rates derived from routine DSS reports. Similar findings have been reported from other places in Tanzania [32]. Estimates of entomological inoculation rates (EIR) in the Kilombero Valley did not reveal elevated malaria transmission in the *shamba *compared to village locations [15]. Lower malaria transmission fever incidence in the *shamba *could be attributed to lower population density in the fields and different feeding behaviour and transmission capacity of vectors, potentially leading to less transmission [4]. Yet, it may also be influenced by human behaviour. As *shamba *houses are very scattered, there is little opportunity for social life after dusk. People would, therefore, retreat to their huts and go to bed earlier than in the villages and with the high mosquito net usage rates, most people would be effectively protected against *Anopheles *bites. Other explanation for the lower fever incidence in the *shamba *could not be found.

The analysis of treatment-seeking behaviour showed that most fever episodes were treated with an antimalarial and 56.9% of the episodes reached a health facility at some stage. Despite the long distance to the nearest facilities, few episodes were exclusively managed at home. This is supported by the findings of a related study which found that episodes from even the most distant *shamba *were eventually treated at a health facility [27]. Similar to the ITN usage rates, the frequent use of biomedical treatment may in part be attributed to continuous and intensive malaria control activities. Hence, in areas with less intensive control activities, different treatment-seeking patterns may be observed. No significant differences could be seen between episodes which were first recognized at the *shamba *or at the main house. This may on one hand be due to the small sample size. On the other hand, it was often observed that in case of an illness episode, household moved back to their main house from where treatment-seeking action would then be started.

From this study, the considerable distance between farming sites and health services did not appear to lead to a delay in treatment-seeking. However, the use of quantitative methods – a semi-structured questionnaire – may also have influenced this result. Mayumana [27] investigated treatment delay in more detail in a qualitative study on livelihoods and health care. It appeared from his study that most cases occurring in the families' main houses were treated within 24 hours while episodes in the *shamba *only after three to five days.

Surprisingly, home management was less common the further away the nearest health facility was. This may be related to the fact that few households stocked antimalarial drugs in the *shamba *and households far from health facilities were also far away from drug stores or general shops stocking drugs. Hence, there was little opportunity to purchase antimalarials from alternative providers. Nevertheless, home management was still more frequent in this *shamba *survey (37.5%) than in a cross-sectional community survey done in 2004 in the same area. In that study, 23.4% of fever episodes were exclusively treated at home. At the same time, health facility attendance was higher with 76% children under 5 years of age and 56% adults being brought to a health facility [23]. This supports the assumption that access to health facilities is in fact more difficult for families staying in the fields compared to the population in general. Episodes which occur in the *shamba *would, therefore, demand greater efforts (in terms of money and time) for families to obtain the preferred treatment and care at health facilities. Home-management with drugs provided to the households in or close to the *shamba *would most likely facilitate timely access to appropriate treatment but would need to be coupled with education of care-takers and training of drug-providers [31]. However, any implementation strategy would have to take into account the draw-backs of low population densities and widely dispersed households in the fields.

## Conclusion

Seasonal movements of households to farming sites located at a considerable distance from their main residences are very common in the Kilombero Valley. Despite high malaria endemicity in this area, no increased fever incidence could be detected in people who spend days and nights in temporary shelters on their rice fields. Individual protection with mosquito nets was very high but preparedness for malaria episodes rather poor. Inability to obtain antimalarials easily from nearby drugs stores forced people to seek help from distant health facilities. On the one hand, this may facilitate the implementation of good quality case-management and rational prescription of drugs. However, each episode occurring in the *shamba *may well become a heavier social and economic burden than under normal circumstances. In such a situation, a comprehensive approach is needed to improve timely access to affordable treatment and care, while at the same time assuring rational use of medicines.

## Authors' contributions

MWH designed the study, developed the survey tools, participated in the data analysis and co-wrote the manuscript. SA participated in the data analysis and co-wrote the manuscript. MF supervised the data collection and participated in data analysis. IM coordinated the data collection, data cleaning and contributed to the manuscript. CL, BO, AS and HM conceived the study, provided technical support and contributed to the manuscript. RN provided the DSS data. AM and CM contributed to the discussion on the manuscript and facilitated the field work.
